# Regulation of A disintegrin and metalloproteinase (ADAM) family sheddases ADAM10 and ADAM17: The emerging role of tetraspanins and rhomboids

**DOI:** 10.1080/09537104.2016.1184751

**Published:** 2016-06-02

**Authors:** Alexandra L. Matthews, Peter J. Noy, Jasmeet S. Reyat, Michael G. Tomlinson

**Affiliations:** ^a^ School of Biosciences, College of Life and Environmental Sciences, University of Birmingham, Birmingham, UK

**Keywords:** ADAM10, ADAM17, iRhom, platelet, tetraspanin, TspanC8

## Abstract

A disintegrin and metalloprotease (ADAM) 10 and ADAM17 are ubiquitous transmembrane “molecular scissors” which proteolytically cleave, or shed, the extracellular regions of other transmembrane proteins. ADAM10 is essential for development because it cleaves Notch proteins to induce Notch signaling and regulate cell fate decisions. ADAM17 is regarded as a first line of defense against injury and infection, by releasing tumor necrosis factor α (TNFα) to promote inflammation and epidermal growth factor (EGF) receptor ligands to maintain epidermal barrier function. However, the regulation of ADAM10 and ADAM17 trafficking and activation are not fully understood. This review will describe how the TspanC8 subgroup of tetraspanins (Tspan5, 10, 14, 15, 17, and 33) and the iRhom subgroup of protease-inactive rhomboids (iRhom1 and 2) have emerged as important regulators of ADAM10 and ADAM17, respectively. In particular, they are required for the enzymatic maturation and trafficking to the cell surface of the ADAMs, and there is evidence that different TspanC8s and iRhoms target the ADAMs to distinct substrates. The TspanC8s and iRhoms have not been studied functionally on platelets. On these cells, ADAM10 is the principal sheddase for the platelet collagen receptor GPVI, and the regulatory TspanC8s are Tspan14, 15, and 33, as determined from proteomic data. Platelet ADAM17 is the sheddase for the von Willebrand factor (vWF) receptor GPIb, and iRhom2 is the only iRhom that is expressed. Induced shedding of either GPVI or GPIb has therapeutic potential, since inhibition of either receptor is regarded as a promising anti-thrombotic therapy. Targeting of Tspan14, 15, or 33 to activate platelet ADAM10, or iRhom2 to activate ADAM17, may enable such an approach to be realized, without the toxic side effects of activating the ADAMs on every cell in the body.

## Introduction

**Figure 1.**
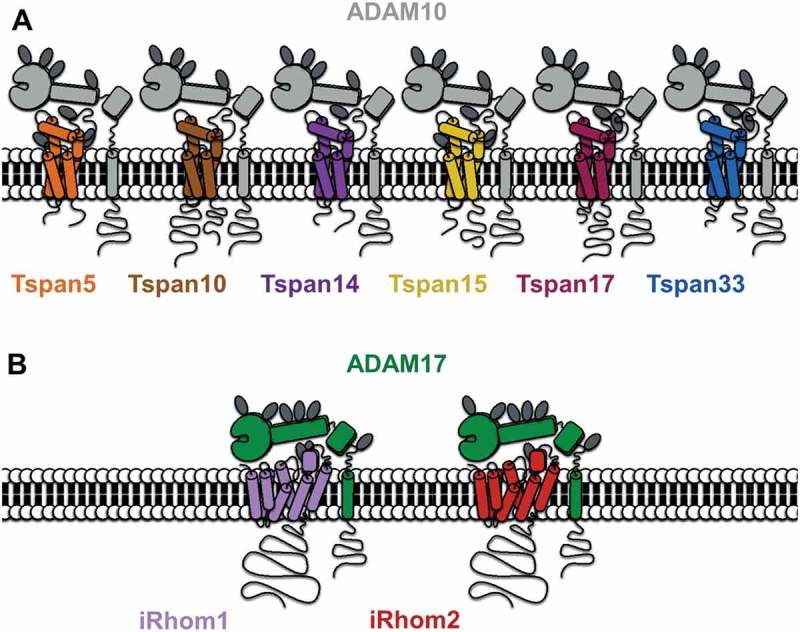
A diagrammatic representation of ADAM10 interacting with each of six TspanC8s (A) and ADAM17 interacting with each of iRhom1 and 2 (B). Note that the TspanC8s differ in the number of N-linked glycosylation sites (dark gray ovals) and in the lengths of their cytoplasmic tails (the Tspan5 C-terminus is shortest at 15 amino acids and the Tspan10 N-terminus is longest at 77 amino acids). The iRhoms are relatively similar but differ in N-glycosylation.

Protein cleavage, or “shedding,” of the extracellular regions (ectodomains) of transmembrane proteins has emerged as a key regulatory process in cell biology. “Sheddases” can modulate signaling on host or neighboring cells either directly through the regulation of cell surface receptors or indirectly through the release of soluble mediators from their membrane-bound precursors [1]. The ADAMs (A disintegrin and metalloproteinases) are one of the major proteinase families that function as sheddases. They are characterized by a modular domain structure, which is comprised of an N-terminal signal sequence followed by a prodomain, metalloprotease domain, disintegrin domain, cysteine-rich region, EGF-like domain (not in ADAM10 and 17), transmembrane domain, and a cytoplasmic tail (Figure 1). The ADAM family consists of 22 members, as identified in the human genome, of which only 12 (ADAM8, 9, 10, 12, 15, 17, 19, 20, 21, 28, 30, and 33) encode active enzymes [2].

## ADAM10 and ADAM17: ubiquitous and essential “molecular scissors”

ADAM10 (also known as Kuzbanian from its orthologous gene, *kuz*, in *Drosophila*) and its close relative ADAM17 (also recognized as tumor necrosis factor α (TNFα) converting enzyme or TACE) are ubiquitously expressed in mammalian cells. ADAM10 and ADAM17 share 30% amino acid identity in human and are the principal ADAM sheddases; they are capable of cleaving numerous substrates with diverse functions, whereas other ADAMs appear to have a more restricted substrate range [3,4].

ADAM10 has at least 40 substrates, many of which have important roles in health and disease. These include the Notch cell fate regulators [5–8], amyloid precursor protein (APP) [9], cellular prion protein [10], epidermal growth factor receptor (EGFR) ligands betacellulin and EGF [11], adhesion molecules E-cadherin [12], N-cadherin [13], VE-cadherin [14] and CD44 [15], transmembrane chemokines CX3CL1 and CXCL16 [16], the low-affinity immunoglobulin E receptor CD23 [17], and vascular endothelial growth factor receptor 2 [18]. ADAM10-deficient mice phenocopy Notch-deficient mice; they die at embryonic day 9.5 with multiple defects of the somites, cardiovascular, and neuronal systems [19]. This demonstrates the key role of ADAM10 in the activation of Notch proteins. ADAM10 appears to have constitutive activity toward some substrates, but its activity can be upregulated by certain stimuli that induce intracellular signaling. The activation mechanism is not clear, but the ADAM10 transmembrane region, and not the cytoplasmic tail, is important in this process [20]. There is also evidence that intracellular signaling renders substrates more susceptible to cleavage, for example following phosphorylation of the CD44 cytoplasmic tail. This may then induce conformational changes and/or dimerization in the CD44 extracellular region, to promote cleavage by ADAM10 [21,22].

ADAM17 appears to have at least as many substrates as ADAM10. It is rapidly activated by a great variety of stimuli to induce the shedding and release of soluble TNFα and the EGFR ligands amphiregulin, epiregulin, heparin-binding EGF-like growth factor, and transforming growth factor α (TGFα) [23]. Therefore, ADAM17 has been proposed to function as a first line of defense against injury, by promoting inflammation and repair of skin and intestine barrier function. Consistent with such a function, ADAM17-deficient mice phenocopy EGFR-deficient mice; they die shortly after birth and have skin barrier defects, open eyes at birth, enlarged heart valves, abnormal mammary ductal morphogenesis, and lung defects [24–28]. Other ADAM17 substrates include the adhesion molecules ICAM-1 [29] and L-selectin [28], and TNF receptor family members TNFRI [30] and TNFRII [28]. Several studies have investigated the mechanism by which ADAM17 activity is regulated. Similar to ADAM10, there is evidence that activation of ADAM17 is dependent on its transmembrane domain but not the cytoplasmic tail [23]. However, a second study provides evidence that the cytoplasmic tail is important in negatively regulating ADAM17 [31]. The cytoplasmic tail promotes dimerization, which also appears to be a feature of the ADAM10 tail [31,32]. Phosphorylation of the ADAM17 tail, by the mitogen-activated protein kinases (MAPKs) extracellular signal-regulated kinase (Erk) and p38, reduces dimerization, which decreases tissue inhibitor of metalloproteinase-3 (TIMP3) interaction with the extracellular region of ADAM17 and promotes activation [31]. Another reported regulatory mechanism involves direct inhibition of the ADAM17 extracellular region by protein disulfide isomerize [33–35]. The latter can interact with ADAM17 and alter the disulfide bonding within the membrane-proximal cysteine-rich region. This appears to convert the ADAM17 extracellular region from an extended “open” active conformation into a “closed” inactive conformation [33]. Finally, the lipid second messenger ceramide 1-phosphate, a product of ceramide kinase, can interact with ADAM17 and inhibit its activity by an as yet undefined mechanism [36].

On platelets, ADAM10 is the major sheddase for GPVI [37,38], the main collagen-activated receptor which has recently been shown to also bind fibrin [39,40]. ADAM17 is a sheddase for GPIbα [41] and GPV [42] of the GPIb–IX–V complex, which has a variety of ligands including von Willebrand factor (vWF), thrombin, coagulation factors XI and XII, P-selectin, and the leukocyte integrin αMβ2 [43]. GPIb-IX-V is essential for hemostasis and GPVI has a minor role, but both are potential anti-platelet drug targets because they promote thrombosis [44]. The physiological role of their shedding is not clear, since mice with platelets deficient in ADAM10, ADAM17, or both have normal platelet size and count [37]. However, ADAM17 appears to constitutively shed GPIbα, since mice with ADAM17-knockout platelets have reduced plasma levels of glycocalicin, a soluble fragment of GPIbα [37]. Shedding may be important in disease processes. For example, *Staphylococcus aureus* α-toxin binds to and activates ADAM10 [45], one consequence of which is shedding of GPVI which contributes to lethal sepsis in mice [46]. Furthermore, soluble GPVI is elevated in human patients in several diseases, including coronary artery disease and ischemic stroke [47]. ADAM10 and ADAM17 have some substrates in common, and there is evidence that ADAM10 can shed GPV and that ADAM17 sheds GPVI [37,38]. Indeed, expression levels of GPVI and GPIb are mildly elevated on ADAM10/17 double-deficient platelets, but not on single-knockout platelets [37]. The importance of ADAM10/17 shedding of other substrates in the platelet/megakaryocyte lineage is not known. The additional ADAM10 substrates include Notch proteins, APP, CD44, EGF, and CD84, although only the latter has been confirmed as an ADAM10 substrate on platelets and its function is not clear [48]. An additional ADAM17 substrate on platelets is the semaphorin Sema4D, which binds to plexin family receptors and amplifies GPVI-induced platelet activation [49,50].

The ubiquitous expression of ADAM10 and ADAM17 and full repertoire of their substrates is reflected in the pleiotropic function of these enzymes, with roles in cell adhesion, proliferation, differentiation, migration, immunity, and receptor-ligand signaling [3,4]. However, the mechanisms by which ADAM10 and ADAM17 activity are regulated are not fully understood. This review will cover the emerging role of two families of multi-transmembrane proteins in the regulation of ADAM10 and ADAM17, namely the tetraspanins and rhomboids, respectively.

## Regulation of ADAM10 by tetraspanins

### Tetraspanins regulate the trafficking and membrane dynamics of other transmembrane proteins

The tetraspanins are an evolutionarily conserved superfamily of 33 transmembrane proteins in mammals, which extends to flies, worms, multicellular fungi, and plants [51,52]. Tetraspanins contain four transmembrane domains that delineate two extracellular loops of unequal size (the larger of which has four to eight conserved cysteine residues that form structurally important disulfide bonds), one intracellular loop, and intracellular N- and C-termini (Figure 1). Structural studies have revealed that tetraspanins fold to form compact, rod-shaped structures that protrude 3–5 nm from the plasma membrane [53]. Tetraspanins are not generally believed to have ligands or to function as cell surface receptors, but instead they are thought to self-associate with one another and with their so-called “partner” proteins into nanoclusters. Tetraspanin partner proteins include ADAM10, integrins, and members of the immunoglobulin superfamily [51,52].

A recent super-resolution microscopy study revealed that most tetraspanin nanoclusters appear to contain only one type of tetraspanin [54]. This helps to explain the specific functions that have been assigned to distinct tetraspanins through gene-knockout studies. These include the role of tetraspanin CD151 in regulating its laminin-binding integrin partner proteins α3β1, α6β1, and α6β4 [52]. Indeed, CD151 promotes adhesion strengthening of cells to laminin [55], integrin recycling from the plasma membrane [56,57], and glycosylation of α3 during biosynthesis [58] and restricts the lateral mobility of α6 in the plasma membrane [59]. Tetraspanin CD81 regulates glycosylation of the B cell signaling protein CD19 and is essential for its trafficking to the plasma membrane [60]. On endothelial cells, tetraspanin Tspan12 promotes Frizzled 4 clustering and signaling in response to its ligand norrin [61], while tetraspanins CD9, CD151, and CD63 promote inflammatory leukocyte capture by clustering adhesion molecules ICAM-1, VCAM-1 [62], and P-selectin [63], respectively. Importantly, these findings are all supported by tetraspanin-knockout or -knockdown studies, in which the tetraspanin knockout phenocopies that of the partner protein. Moreover, human mutations in CD151, CD81, and Tspan12 cause diseases that are consistent with functional impairment of the respective partners [64–67].

In platelets, tetraspanin-deficient mice have revealed a positive role for CD151 and Tspan32 in hemostasis, which is proposed to be mediated by their regulation of outside-in signaling by the major platelet integrin αIIbβ3 [68,69]. However, this is likely to be a fine-tuning role, or an indirect effect, because the CD151 and Tspan32-knockout phenotypes are less severe than the αIIbβ3 knockout, and their copy numbers on platelets are several-fold lower than the integrin [70–72]. CD82 plays an opposing role to CD151 and Tspan32, since CD82-deficient mice have reduced bleeding times and enhanced clot retraction [73]. Surface expression levels of αIIbβ3 are approximately 30% elevated in the absence of CD82 [73], but again regulation of the integrin is unlikely to be direct, because of the relatively low CD82 copy number [72]. Platelets deficient in CD9 or CD63 have subtle defects, suggesting relatively minor roles for these tetraspanins in hemostasis [74,75].

### The TspanC8 subgroup of tetraspanins interact with ADAM10 and promote its exit from the endoplasmic reticulum and enzymatic maturation

**Figure 2.**
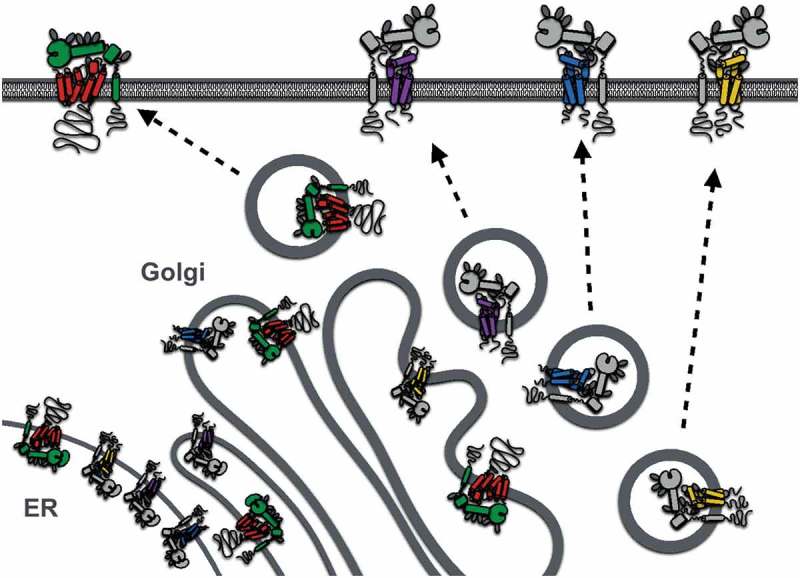
Regulation of ADAM10 and ADAM17 exit from the endoplasmic reticulum (ER), enzymatic maturation, and trafficking to the plasma membrane by TspanC8s and iRhoms, respectively. Only Tspan14, 15, 33, and iRhom2 are depicted because of their expression in human platelets. Enzymatic maturation involves cleavage of the ADAM prodomain in the Golgi by proprotein convertases.

The first clues to ADAM10 regulation by tetraspanins were the findings that it co-immunoprecipitates with tetraspanins in human leukocyte cell lines and that tetraspanin antibodies promote ADAM10 activity, as measured by the shedding of ADAM10 substrates such as EGF [76]. Subsequently, we and others showed that six largely unstudied tetraspanins (Tspan5, 10, 14, 15, 17, and 33) co-immunoprecipitate with ADAM10 in stringent lysis buffers, whereas other tetraspanins do not [77,78]. The ADAM10-interacting tetraspanins are related by amino acid sequence, with the human homologs ranging from 78% amino acid identity between Tspan5 and 17 to 26% identity between the most distantly related Tspan10 and 15. These tetraspanins were termed the TspanC8 subgroup due to the eight cysteine residues that they possess within their large extracellular loop [77,78]; other tetraspanins have four, six, or seven. An unrelated tetraspanin, Tspan12, was also reported to regulate ADAM10 [79], but this is now thought to be an indirect effect [77,78], potentially mediated via Tspan12 interaction with, and/or regulation of, TspanC8 tetraspanins. Overexpression of any of the six TspanC8s in cell lines promotes ADAM10 exit from the endoplasmic reticulum (ER) and its enzymatic maturation [77, 78, 80] (Figure 2). This is the process by which the prodomain is removed by proprotein convertases, such as Furin, in the Golgi to unmask the catalytic site [81]. Different cell types have their own TspanC8 repertoires [78]. Knockdown or knockout of the most highly expressed TspanC8, in several primary cells and cell lines that have been studied, prevents ADAM10 ER exit and trafficking to the plasma membrane [77, 78, 80]. Importantly, TspanC8-knockout mice and flies yield phenotypes that are consistent with these data. In mice, Tspan33 is the predominant TspanC8 in the red blood cell lineage; Tspan33 deficiency in mice results in impaired red blood cell production and substantially reduced ADAM10 expression on red cells [78]. In *Drosophila*, there are three TspanC8s, and these are required for Notch activity and normal fly development [77], consistent with the key role for ADAM10 in Notch activation. Taken together, these studies demonstrate an essential role for the TspanC8s in promoting ADAM10 intracellular trafficking (Figure 2).

### ADAM10 “six appeal”: evidence that ADAM10 can be regarded as six different molecular scissors, depending upon which TspanC8 it is partnered with

An important question arising from the aforementioned studies is why six TspanC8 tetraspanins have evolved to regulate ADAM10 trafficking. One theory is that each of the six TspanC8 tetraspanins may target ADAM10 to distinct subcellular localizations and/or substrates. Different TspanC8s certainly appear to have different subcellular localizations when transfected into cell lines, with some promoting ADAM10 trafficking to intracellular compartments and others to the plasma membrane [77]. Moreover, there is growing evidence to suggest that the different TspanC8s can promote or suppress the ADAM10-mediated shedding of specific substrates. For example, Tspan5, Tspan10, and Tspan14 appear to be positive regulators of Notch signaling, whereas Tspan15 and Tspan33 are negative regulators [77, 82, 83]. Similarly interesting data have been reported for other ADAM10 substrates: N-cadherin cleavage is specifically promoted by Tspan15 [80, 82, 84], APP cleavage is inhibited by Tspan15 in U2OS and PC3 cells [82], but promoted by this tetraspanin in HEK and N2A cells [80], and CD44 cleavage is promoted by Tspan5 but not Tspan15 [82]. These studies each used TspanC8 overexpression or knockdown in cell lines, and some differences were observed between different cells, thus complicating the interpretations. This likely reflects the different endogenous TspanC8 repertoires, and potentially associated trafficking proteins, of each cell type. There is scope for the cytoplasmic tails of TspanC8s to interact with specific trafficking proteins, since these tails show no substantial sequence similarity between different TspanC8s. They are also relatively long for tetraspanins, with N- and C-termini having average lengths of 29 and 36 amino acids, respectively, which are two to three times longer than is typical for most other tetraspanins. However, TspanC8 interactions with trafficking proteins have yet to be identified.

To begin to determine the mechanisms responsible for different subcellular localization and shedding by distinct TspanC8–ADAM10 complexes, their lateral mobility and associated proteins have been investigated [82]. ADAM10 single-particle tracking studies using total internal reflectance fluorescence microscopy suggest that Tspan15 promotes ADAM10 lateral mobility at the plasma membrane, but Tspan5 does not. In addition, proteomic studies identified a number of proteins that preferentially co-immunoprecipitate with Tspan5 versus Tspan15 [82]. Furthermore, we have used mutant forms of TspanC8s and ADAM10 to show that the TspanC8–ADAM10 interaction is largely mediated by the main extracellular region of the TspanC8. This binds to the membrane-proximal stalk, disintegrin, and cysteine-rich domain regions of ADAM10 [84]. However, there are some differences between TspanC8s in the precise binding regions that they require on ADAM10. This suggests that ADAM10 may adopt a different conformation, depending on which TspanC8 it is associated with [84]. This may in part dictate substrate specificity. An additional possibility is that TspanC8s target ADAM10 to a substrate by directly binding to the substrate. There is no evidence for this at present, but it is interesting that some ADAM10 substrates, including GPVI and CD44, have been reported to interact with an as yet unidentified tetraspanin [71, 85].

**Figure 3.**
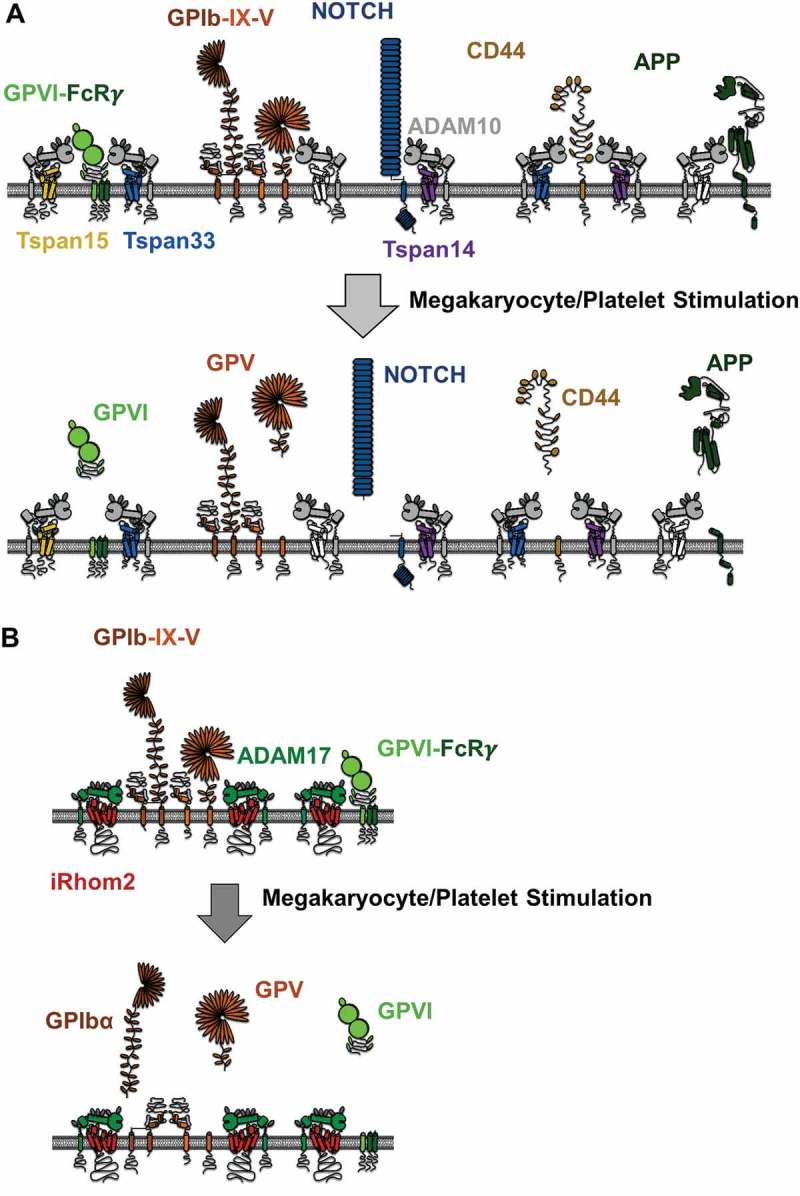
ADAM10 and ADAM17 substrate cleavage on the megakaryocyte/platelet surface. (A) GPVI is not constitutively cleaved on platelets but is cleaved upon platelet activation. Some other ADAM10 substrates, GPV, Notch, CD44, and amyloid precursor protein (APP) are depicted, but their cleavage on platelets is not well characterized. Nevertheless, studies on other cell types, and in cell line models, suggest that different TspanC8s promote cleavage of distinct substrates. For example, Tspan14 appears to protect GPVI from cleavage, Tspan14 promotes Notch cleavage but Tspan15 inhibits, Tspan15 cannot promote CD44 cleavage, and Tspan15 effects on APP are cell type dependent. (B) GPIbα is constitutively cleaved by ADAM17. GPV and GPVI also appear to be shed by ADAM17 following platelet activation. Studies in other cell types suggest that ADAM17 trafficking and activation may be regulated by iRhom2, but this has yet to be tested in megakaryocytes or platelets.

ADAM10 substrates on platelets include GPVI, GPV, APP, CD44, CD84, and EGF. It has yet to be investigated whether specific TspanC8s promote or suppress shedding of any of these substrates on platelets, although speculations can be made based on studies in other cell types (Figure 3). However, investigating such speculations will be difficult because it is not possible to genetically modify the anucleate platelet directly, and knockout mice are only available for Tspan33, which does not appear to be expressed in mouse platelets [78, 86]. Furthermore, monoclonal antibodies have yet to be generated for any TspanC8. Quantitative proteomics of human and mouse platelets [86, 87] nevertheless allow TspanC8 copy numbers to be estimated (Table I). Human platelets express similar levels of Tspan14, 15, and 33 [87], whereas mouse platelets only express Tspan14 [86]. The latter is consistent with our quantitative PCR analyses of mouse megakaryocytes, which show Tspan14 to be the most highly expressed TspanC8 in these cells [78]. This suggests a more limited scope for ADAM10 regulation by TspanC8s in mouse platelets. Nevertheless, we have detected Tspan5, 17, and 33 mRNA in mouse megakaryocytes, albeit at relatively low levels [71, 78], indicating that these additional TspanC8s might regulate ADAM10 during megakaryocyte development. In keeping with their intimate relationship, the total TspanC8 protein copy number is comparable to the ADAM10 copy number in both human and mouse platelets (Table I).

**Table I.** ADAM10 and ADAM17 are expressed at a similar copy number to their regulatory TspanC8s and iRhoms, in human and mouse platelets.

Protein	Human platelet copy number (Burkhart et al. 2012)	Mouse platelet copy number (Zeiler et al. 2014)
ADAM10	4400	9889
ADAM17	670	151
iRhom1	0	0
iRhom2	1000	293
Tspan5	0	0
Tspan10	0	0
Tspan14	2000	8664
Tspan15	2500	0
Tspan17	0	0
Tspan33	2100	0

Data were obtained from quantitative proteomic studies [86,87].

We have confirmed Tspan14 expression in human and mouse platelets by the generation of a polyclonal western blotting antibody, and we have further confirmed its interaction with ADAM10 on these cells [84]. To investigate whether TspanC8s might affect GPVI shedding, we have adopted a cell line transfection model. Strikingly, Tspan14 overexpression suppresses ADAM10 cleavage, but the other TspanC8s have no effect [84]. We speculate that Tspan14 functions to protect GPVI from ADAM10 shedding on resting platelets, but may be induced to promote shedding, or release its inhibitory effect on ADAM10, upon platelet activation. This would help to explain why GPVI is not cleaved in resting platelets, but is rapidly cleaved in response to various stimuli including its physiological ligand collagen, shear stress, activated coagulation factor Xa, GPVI antibodies, and other platelet-activating agonists [47]. Since GPVI is regarded as a promising anti-platelet drug target to treat thrombosis [44], the induction of GPVI shedding by targeting the Tspan14–ADAM10 complex has some potential.

Taken together, emerging evidence indicates that different TspanC8s regulate shedding of distinct ADAM10 substrates. This may be due to effects on ADAM10 subcellular localization, conformation, and/or direct TspanC8–substrate interactions. This leads us to suggest that ADAM10 should not be regarded as a single molecular scissor, but instead can exist as one of six different scissors, depending on which TspanC8 it is partnered with.

## Regulation of ADAM17 by rhomboids

### The rhomboid superfamily of intramembrane proteases

The rhomboids, like the tetraspanins, are an evolutionarily conserved superfamily of multi-transmembrane proteins [88, 89]. However, rhomboids have six or seven transmembrane domains and are even more widespread than tetraspanins, being present in bacteria as well as animals, plants, and fungi. Rhomboids are serine proteases (or catalytically inactive non-proteases), which are characterized by the presence of the active protease site within the membrane, where transmembrane substrates are cut [90]. The founding member of the rhomboid superfamily is *Drosophila* rhomboid-1, which releases ligands for the EGF receptor. The five mammalian active rhomboids are RHBDL1-4 and PARL. RHBDL1-4 are expressed on the secretory pathway and are not well understood, with definitive substrates yet to be identified. RHBDL1-3 may promote the release of secreted proteins, while RHBDL4 is ER localized and may regulate ER-associated degradation, which is a quality-control process to remove incorrectly folded proteins. In contrast, PARL is relatively well studied and important for mitochondrial structure and function, with potential roles in type 2 diabetes and Parkinson’s disease [88, 89]. The non-protease rhomboids lack essential residues within the catalytic site that are required for protease activity. There are nine non-protease rhomboids in mammals. Of these, derlins 1–3 appear to regulate ER-associated degradation, while RHBDD2, RHBDD3, TMEM115, and UBAC2 are relatively poorly understood [88, 89]. The other two non-protease rhomboids are iRhom1 and iRhom2, which have recently emerged as regulators of ADAM17 [91–93] and will be the focus of the remainder of the review.

### iRhom1 and 2 interact with ADAM17 and promote its exit from the ER and enzymatic maturation

Amongst the non-protease rhomboids, the iRhoms are the most closely related by amino acid sequence to the active rhomboids, and they are the most studied [88, 89]. The two human iRhoms share 61% amino acid identity and are characterized by seven transmembrane domains and a relatively large cytoplasmic N-terminus of approximately 410 amino acids (Figure 1). A function for iRhoms was first identified in *Drosophila*, where it was shown that the single iRhom in this organism counteracts the function of active rhomboid-1 [94]. *Drosophila* iRhom achieves this by interacting with transmembrane EGFR ligands and targeting them to the ER-associated degradation pathway, before they can be released as active ligands by rhomboid-1 [94].

In mammals, however, this is not the role for iRhom1 and 2, since the generation of iRhom2-deficient or mutant mice demonstrated that this iRhom is essential for release of the inflammatory cytokine TNFα from macrophages [91–93]. As a result, iRhom2-deficient mice have substantially reduced serum levels of TNFα upon inflammatory challenge with bacterial lipopolysaccharide and consequently reduced survival upon infection with the intracellular bacterium *Listeria monocytogenes* [92]. As described earlier, ADAM17 is the sheddase for TNFα, and iRhom2 was shown to interact with ADAM17 in the ER and to promote its enzymatic maturation and trafficking to the cell surface [91, 92] (Figure 2). Consistent with such a regulatory role, the iRhom2-deficient mice phenocopy myeloid-specific ADAM17-deficient mice in an inflammatory arthritis model, in which both strains are protected [95]. The macrophage phenotype observed in iRhom2-deficient mice is consistent with the fact that iRhom2 expression is restricted to certain hematopoietic cells such as macrophages, whereas iRhom1 is more widely expressed but is not expressed in macrophages [95, 96]. This is particularly exciting from an anti-inflammatory therapeutic angle. Targeting iRhom2 may reduce inflammation by specifically preventing TNFα release from macrophages, without the side effects of the widely used global TNFα-targeting drugs.

A recent characterization of iRhom1/2 double-deficient mice suggests that the primary role of iRhoms is to regulate ADAM17, since the phenotype is a remarkable phenocopy of ADAM17-deficient mice [97]. In particular, the mice are born with open eyes and die shortly after birth. Furthermore, ADAM17 maturation and EGFR phosphorylation are almost completely absent in a panel of iRhom1/2 double-deficient tissues, suggesting an absence of ADAM17 activity [97]. However, an earlier study suggested that iRhoms have functions in addition to their regulation of ADAM17, since a different iRhom1/2-deficient mouse model has a more severe phenotype, which results in embryonic lethality between days 9.5 and 10.5 [96]. This discrepancy appears to be due to differences in iRhom1 single-knockout phenotypes between the two models. Indeed, in the more recent study, iRhom1-deficient mice are found to be viable, albeit with impaired ADAM17 maturation in brain tissue [97]. In the earlier study, however, iRhom1-deficient mice died between 9 days and 6 weeks after birth with defects in multiple tissues [96]. It therefore remains unclear whether iRhoms have roles in addition to their regulation of ADAM17.

The promotion of ADAM17 maturation and trafficking to the cell surface by iRhoms appears to extend to humans. Dominant active iRhom2 mutations, within the N-terminal cytoplasmic tail, cause a rare condition known as tylosis with esophageal cancer, characterized by hyperproliferation of epidermal cells, leading to thickening of the skin on palms and soles, and increased susceptibility to cancer of the esophagus [98,99]. This is associated with increased ADAM17 maturation and activity on keratinocytes, increased release of EGFR ligands, and increased EGFR signaling [100]. Interestingly, ADAM17 deficiency has been identified in two siblings and is the likely cause of autosomal recessive neonatal inflammatory skin and bowel lesions [101]. Unlike in mice, both survived into childhood, and one is now an adult with only repeated skin infections as a symptom [101]. Nevertheless, both studies are consistent with an important role for iRhom-regulated ADAM17 in epidermal barrier function.

### Could iRhom1/ADAM17 and iRhom2/ADAM17 be regarded as two different molecular scissors?

Both iRhom1 and 2 can interact with ADAM17 and promote its maturation in a transfected cell line [96]. Both are similarly activated in a cell line by partial truncations of the N-terminal cytoplasmic tail, each resulting in increased ADAM17 activity toward two of its substrates that were tested, TNFR1 and TNFR2 [102]. However, evidence that iRhoms promote the shedding of distinct ADAM17 substrates has been reported. In a mouse embryonic fibroblast model for ADAM17-mediated shedding of a variety of transfected substrates, iRhom2 is critical for the shedding of heparin-binding EGF-like growth factor, epiregulin, EphB4, Tie2, and Kit ligand 2, and shedding of the latter is unaffected by the presence or absence of iRhom1 [103]. TGFα shedding appears to be promoted by both iRhom1 and iRhom2, whereas ICAM-1 and L-selectin shedding is independent of iRhom2, and so may be promoted by iRhom1 [103]. These differential iRhom effects on ADAM17 shedding may be dependent on cell type, since L-selectin shedding is dependent on iRhom2 but not iRhom1 in leukocytes [97], which contrasts with the findings reported in mouse embryonic fibroblasts [103]. In mouse embryonic fibroblasts, the N-terminal cytoplasmic tail of the iRhoms is essential for phorbol ester-induced ADAM17 shedding [103]. The authors speculate that a key role for the N-terminal cytoplasmic region, in registering an intracellular signal and somehow inducing activity of its associated ADAM17, could explain their earlier observation that the cytoplasmic tail of ADAM17 is dispensable for induced activation [23,103]. Taken together, it appears likely that iRhom1 and 2 enable ADAM17 to function as two distinct molecular scissors, with different substrate specificities and potentially different activating stimuli. However, given that there are only two iRhoms, there is substantially less scope for differential regulation of ADAM17 than for the regulation of ADAM10 by the six TspanC8s.

The iRhoms are functionally unstudied in platelets. Quantitative proteomic studies suggest that iRhom2, but not iRhom1, is expressed by platelets [86,87]. Moreover, the estimated iRhom2 copy number is remarkably similar to that of ADAM17 in human and mouse platelets (Table 1). As such, iRhom2 would be predicted to regulate ADAM17 maturation and trafficking to the cell surface in megakaryocytes, with the result that ADAM17 expression may be deficient in iRhom2-knockout platelets.

## Conclusions

Within the last 4 years, two distinct types of multi-transmembrane proteins have emerged as critical regulators of ADAM10 and ADAM17: the TspanC8 subgroup of tetraspanins and the iRhom subgroup of inactive rhomboids, respectively. Accumulating evidence suggests that ADAM10 and ADAM17 can be regarded as different types of molecular scissor, depending on which TspanC8 or iRhom they are associated with. Further cell biology research is now required to identify which substrates are preferentially shed by which ADAM10–TspanC8 or ADAM17–iRhom complex and to determine the mechanisms that are responsible for such preferences. For platelet biologists, the platelet-expressed TspanC8s (Tspan14, 15, and 33) represent potential therapeutic targets for the activation of ADAM10, with the aim of shedding the pro-thrombotic collagen receptor GPVI from the platelet surface. Similarly, iRhom2 targeting holds anti-thrombotic potential, by promoting ADAM17-induced shedding of GPIb.

## References

[1] Khokha R, Murthy A, Weiss A. Metalloproteinases and their natural inhibitors in inflammation and immunity. Nat Rev Immunol 2013;13:649–665. 23969736 10.1038/nri3499

[2] Klein T, Bischoff R. Active metalloproteases of the A Disintegrin and Metalloprotease (ADAM) family: biological function and structure. J Proteome Res 2011;10:17–33. 20849079 10.1021/pr100556z

[3] Dreymueller D, Uhlig S, Ludwig A. ADAM-family metalloproteinases in lung inflammation: potential therapeutic targets. Am J Physiol Lung Cell Mol Physiol 2015;308:L325–43. 25480335 10.1152/ajplung.00294.2014

[4] Saftig P, The Reiss K. “A Disintegrin And Metalloproteases” ADAM10 and ADAM17: novel drug targets with therapeutic potential? Eur J Cell Biol 2011;90:527–35. 21194787 10.1016/j.ejcb.2010.11.005

[5] Bozkulak EC, Weinmaster G. Selective use of ADAM10 and ADAM17 in activation of Notch1 signaling. Mol Cell Biol 2009;29:5679–5695. 19704010 10.1128/MCB.00406-09 PMC2772745

[6] Groot AJ, Habets R, Yahyanejad S, Hodin CM, Reiss K, Saftig P, Theys J, Vooijs M. Regulated proteolysis of NOTCH2 and NOTCH3 receptors by ADAM10 and presenilins. Mol Cell Biol 2014;34:2822–2832. 24842903 10.1128/MCB.00206-14 PMC4135574

[7] Rooke J, Pan D, Xu T, Rubin GM. KUZ, a conserved metalloprotease-disintegrin protein with two roles in Drosophila neurogenesis. Science 1996;273:1227–1231. 8703057 10.1126/science.273.5279.1227

[8] van Tetering G, van Diest P, Verlaan I, van der Wall E, Kopan R, Metalloprotease Vooijs M. ADAM10 is required for Notch1 site 2 cleavage. J Biol Chem 2009;284:31018–1027. 19726682 10.1074/jbc.M109.006775 PMC2781502

[9] Postina R, Schroeder A, Dewachter I, Bohl J, Schmitt U, Kojro E, Prinzen C, Endres K, Hiemke C, Blessing M, et al A disintegrin-metalloproteinase prevents amyloid plaque formation and hippocampal defects in an Alzheimer disease mouse model. J Clin Invest 2004;113:1456–1464. 15146243 10.1172/JCI20864 PMC406531

[10] Altmeppen HC, Prox J, Krasemann S, Puig B, Kruszewski K, Dohler F, Bernreuther C, Hoxha A, Linsenmeier L, Sikorska B, et al The sheddase ADAM10 is a potent modulator of prion disease Elife 2015;4:e04260. 10.7554/eLife.04260 PMC4346534 25654651

[11] Sahin U, Weskamp G, Kelly K, Zhou HM, Higashiyama S, Peschon J, Hartmann D, Saftig P, Blobel CP. Distinct roles for ADAM10 and ADAM17 in ectodomain shedding of six EGFR ligands. J Cell Biol 2004;164:769–779. 14993236 10.1083/jcb.200307137 PMC2172154

[12] Maretzky T, Reiss K, Ludwig A, Buchholz J, Scholz F, Proksch E, de Strooper B, Hartmann D, Saftig P. ADAM10 mediates E-cadherin shedding and regulates epithelial cell-cell adhesion, migration, and beta-catenin translocation. Proc Natl Acad Sci U S A 2005;102:9182–9187. 15958533 10.1073/pnas.0500918102 PMC1166595

[13] Reiss K, Maretzky T, Ludwig A, Tousseyn T, de Strooper B, Hartmann D, Saftig P. ADAM10 cleavage of N-cadherin and regulation of cell-cell adhesion and beta-catenin nuclear signalling. EMBO J 2005;24:742–52. 15692570 10.1038/sj.emboj.7600548 PMC549617

[14] Schulz B, Pruessmeyer J, Maretzky T, Ludwig A, Blobel CP, Saftig P, Reiss K. ADAM10 Regulates Endothelial Permeability and T-Cell Transmigration by Proteolysis of Vascular Endothelial Cadherin. Circ Res 2008;102:1192–1201. 18420943 10.1161/CIRCRESAHA.107.169805 PMC2818019

[15] Anderegg U, Eichenberg T, Parthaune T, Haiduk C, Saalbach A, Milkova L, Ludwig A, Grosche J, Averbeck M, Gebhardt C, et al ADAM10 is the constitutive functional sheddase of CD44 in human melanoma cells. J Invest Dermatol 2009;129:1471–1482. 18971959 10.1038/jid.2008.323

[16] Hundhausen C, Schulte A, Schulz B, Andrzejewski MG, Schwarz N, von Hundelshausen P, Winter U, Paliga K, Reiss K, Saftig P, et al Regulated shedding of transmembrane chemokines by the disintegrin and metalloproteinase 10 facilitates detachment of adherent leukocytes. J Immunol 2007;178:8064–8072. 17548644 10.4049/jimmunol.178.12.8064

[17] Weskamp G, Ford JW, Sturgill J, Martin S, Docherty AJ, Swendeman S, Broadway N, Hartmann D, Saftig P, Umland S, et al ADAM10 is a principal ‘sheddase’ of the low-affinity immunoglobulin E receptor CD23. Nat Immunol 2006;7:1293–1298. 17072319 10.1038/ni1399

[18] Donners MM, Wolfs IM, Olieslagers S, Mohammadi-Motahhari Z, Tchaikovski V, Heeneman S, van Buul JD, Caolo V, Molin DG, Post MJ, et al A disintegrin and metalloprotease 10 is a novel mediator of vascular endothelial growth factor-induced endothelial cell function in angiogenesis and is associated with atherosclerosis. Arterioscler Thromb Vasc Biol 2010;30:2188–2195. 20814017 10.1161/ATVBAHA.110.213124

[19] Hartmann D, de Strooper B, Serneels L, Craessaerts K, Herreman A, Annaert W, Umans L, Lubke T, Lena Illert A, von Figura K, et al The disintegrin/metalloprotease ADAM 10 is essential for Notch signalling but not for alpha-secretase activity in fibroblasts. Hum Mol Genet 2002;11:2615–2624. 12354787 10.1093/hmg/11.21.2615

[20] Maretzky T, Evers A, Le Gall S, Alabi RO, Speck N, Reiss K, Blobel CP. The cytoplasmic domain of a disintegrin and metalloproteinase 10 (ADAM10) regulates its constitutive activity but is dispensable for stimulated ADAM10-dependent shedding. J Biol Chem 2015;290:7416–7425. 25605720 10.1074/jbc.M114.603753 PMC4367251

[21] Hartmann M, Parra LM, Ruschel A, Lindner C, Morrison H, Herrlich A, Herrlich P. Inside-out Regulation of Ectodomain Cleavage of Cluster-of-Differentiation-44 (CD44) and of Neuregulin-1 Requires Substrate Dimerization. J Biol Chem 2015;290:17041–17054. 25925953 10.1074/jbc.M114.610204 PMC4498042

[22] Parra LM, Hartmann M, Schubach S, Li Y, Herrlich P, Distinct Intracellular Herrlich A. Domain Substrate Modifications Selectively Regulate Ectodomain Cleavage of NRG1 or CD44. Mol Cell Biol 2015;35:3381–3395. 26217011 10.1128/MCB.00500-15 PMC4561721

[23] Le Gall SM, Maretzky T, Issuree PD, Niu XD, Reiss K, Saftig P, Khokha R, Lundell D, Blobel CP. ADAM17 is regulated by a rapid and reversible mechanism that controls access to its catalytic site. J Cell Sci 2010;123:3913–3922. 20980382 10.1242/jcs.069997 PMC2972273

[24] Chalaris A, Adam N, Sina C, Rosenstiel P, Lehmann-Koch J, Schirmacher P, Hartmann D, Cichy J, Gavrilova O, Schreiber S, et al Critical role of the disintegrin metalloprotease ADAM17 for intestinal inflammation and regeneration in mice. J Exp Med 2010;207:1617–1624. 20603312 10.1084/jem.20092366 PMC2916135

[25] Franzke CW, Cobzaru C, Triantafyllopoulou A, Loffek S, Horiuchi K, Threadgill DW, Kurz T, van Rooijen N, Bruckner-Tuderman L, Blobel CP. Epidermal ADAM17 maintains the skin barrier by regulating EGFR ligand-dependent terminal keratinocyte differentiation. J Exp Med 2012;209:1105–1119. 22565824 10.1084/jem.20112258 PMC3371728

[26] Jackson LF, Qiu TH, Sunnarborg SW, Chang A, Zhang C, Patterson C, Lee DC. Defective valvulogenesis in HB-EGF and TACE-null mice is associated with aberrant BMP signaling. Embo j 2003;22:2704–2716. 12773386 10.1093/emboj/cdg264 PMC156761

[27] Sternlicht MD, Sunnarborg SW, Kouros-Mehr H, Yu Y, Lee DC, Werb Z. Mammary ductal morphogenesis requires paracrine activation of stromal EGFR via ADAM17-dependent shedding of epithelial amphiregulin. Development 2005;132:3923–3933. 16079154 10.1242/dev.01966 PMC2771180

[28] Peschon JJ, Slack JL, Reddy P, Stocking KL, Sunnarborg SW, Lee DC, Russell WE, Castner BJ, Johnson RS, Fitzner JN, et al An essential role for ectodomain shedding in mammalian development. Science 1998;282:1281–1284. 9812885 10.1126/science.282.5392.1281

[29] Tsakadze NL, Sithu SD, Sen U, English WR, Murphy G, D’Souza SE Tumor necrosis factor-alpha-converting enzyme (TACE/ADAM-17) mediates the ectodomain cleavage of intercellular adhesion molecule-1 (ICAM-1). J Biol Chem 2006;281:3157–64. 16332693 10.1074/jbc.M510797200

[30] Reddy P, Slack JL, Davis R, Cerretti DP, Kozlosky CJ, Blanton RA, Shows D, Peschon JJ, Black RA. Functional analysis of the domain structure of tumor necrosis factor-alpha converting enzyme. J Biol Chem 2000;275:14608–14614:ra34. 10799547 10.1074/jbc.275.19.14608

[31] Xu PL, Liu JM, Sakaki-Yumoto M, Derynck R. TACE Activation by MAPK-Mediated Regulation of Cell Surface Dimerization and TIMP3 Association Science Signaling 2012;5:ra34. 10.1126/scisignal.2002689 PMC4254802 22550340

[32] Deng W, Cho SY, Su PC, Berger BW, Li RH. Membrane-enabled dimerization of the intrinsically disordered cytoplasmic domain of ADAM10. Proceedings of the National Academy of Sciences of the United States of America 2014;111:15987–15992. 10.1073/pnas.1409354111 PMC4234582 25349418

[33] Dusterhoft S, Jung S, Hung CW, Tholey A, Sonnichsen FD, Grotzinger J, Lorenzen I. Membrane-Proximal Domain of a Disintegrin and Metalloprotease-17 Represents the Putative Molecular Switch of Its Shedding Activity Operated by Protein-disulfide Isomerase. J Am Chem Soc 2013;135:5776–5781. 23521534 10.1021/ja400340u

[34] Wang Y, Herrera AH, Li Y, Belani KK, Walcheck B. Regulation of Mature ADAM17 by Redox Agents for L-Selectin Shedding. J Immunol 2009;182:2449–2457. 19201900 10.4049/jimmunol.0802770 PMC2653275

[35] Willems SH, Tape CJ, Stanley PL, Taylor NA, Mills IG, Neal DE, McCafferty J, Murphy G. Thiol isomerases negatively regulate the cellular shedding activity of ADAM17. Biochem J 2010;428:439–450. 20345372 10.1042/BJ20100179

[36] Lamour NF, Wijesinghe DS, Mietla JA, Ward KE, Stahelin RV, Chalfant CE. Ceramide kinase regulates the production of tumor necrosis factor alpha (TNF alpha) via inhibition of TNF alpha-converting enzyme. Journal of Biological Chemistry 2011;286:42808–42817. 22009748 10.1074/jbc.M111.310169 PMC3234830

[37] Bender M, Hofmann S, Stegner D, Chalaris A, Bosl M, Braun A, Scheller J, Rose-John S, Nieswandt B. Differentially regulated GPVI ectodomain shedding by multiple platelet-expressed proteinases. Blood 2010;116:3347–3355. 20644114 10.1182/blood-2010-06-289108

[38] Gardiner EE, Karunakaran D, Shen Y, Arthur JF, Andrews RK, Berndt MC. Controlled shedding of platelet glycoprotein (GP)VI and GPIb-IX-V by ADAM family metalloproteinases. J Thromb Haemost 2007;5:1530–1537. 17445093 10.1111/j.1538-7836.2007.02590.x

[39] Alshehri OM, Hughes CE, Montague S, Watson SK, Frampton J, Bender M, Watson SP. Fibrin activates GPVI in human and mouse platelets. Blood 2015;126:1601–1608. 26282541 10.1182/blood-2015-04-641654 PMC4582337

[40] Mammadova-Bach E, Ollivier V, Loyau S, Schaff M, Dumont B, Favier R, Freyburger G, Latger-Cannard V, Nieswandt B, Gachet C, et al Platelet glycoprotein VI binds to polymerized fibrin and promotes thrombin generation. Blood 2015;126:683–691. 25977585 10.1182/blood-2015-02-629717

[41] Bergmeier W, Piffath CL, Cheng G, Dole VS, Zhang Y, von Andrian UH, Wagner DD. Tumor necrosis factor-alpha-converting enzyme (ADAM17) mediates GPIbalpha shedding from platelets in vitro and in vivo. Circ Res 2004;95:677–683. 15345652 10.1161/01.RES.0000143899.73453.11

[42] Rabie T, Strehl A, Ludwig A, Nieswandt B. Evidence for a role of ADAM17 (TACE) in the regulation of platelet glycoprotein V. J Biol Chem 2005;280:14462–14468. 15691827 10.1074/jbc.M500041200

[43] Bryckaert M, Rosa JP, Denis CV, Lenting PJ. Of von Willebrand factor and platelets. Cell Mol Life Sci 2015;72:307–326. 25297919 10.1007/s00018-014-1743-8 PMC4284388

[44] Metharom P, Berndt MC, Baker RI, Andrews RK. Current state and novel approaches of antiplatelet therapy. Arterioscler Thromb Vasc Biol 2015;35:1327–1338. 25838432 10.1161/ATVBAHA.114.303413

[45] Inoshima I, Inoshima N, Wilke GA, Powers ME, Frank KM, Wang Y, Bubeck Wardenburg J. A Staphylococcus aureus pore-forming toxin subverts the activity of ADAM10 to cause lethal infection in mice. Nat Med 2011;17:1310–1314. 21926978 10.1038/nm.2451 PMC3192248

[46] Powers ME, Becker RE, Sailer A, Turner JR, Bubeck Wardenburg J. Synergistic Action of Staphylococcus aureus alpha-Toxin on Platelets and Myeloid Lineage Cells Contributes to Lethal Sepsis. Cell Host Microbe 2015;17:775–787. 26067604 10.1016/j.chom.2015.05.011 PMC4642999

[47] Gardiner EE, Andrews RK. Platelet receptor expression and shedding: glycoprotein Ib-IX-V and glycoprotein VI. Transfus Med Rev 2014;28:56–60. 24674813 10.1016/j.tmrv.2014.03.001

[48] Hofmann S, Vogtle T, Bender M, Rose-John S, Nieswandt B. The SLAM family member CD84 is regulated by ADAM10 and calpain in platelets. J Thromb Haemost 2012;10:2581–2592. 23025437 10.1111/jth.12013

[49] Mou P, Zeng Z, Li Q, Liu X, Xin X, Wannemacher KM, Ruan C, Li R, Brass LF, Zhu L. Identification of a calmodulin-binding domain in Sema4D that regulates its exodomain shedding in platelets. Blood 2013;121:4221–4230. 23564909 10.1182/blood-2012-11-470609 PMC3656454

[50] Zhu L, Bergmeier W, Wu J, Jiang H, Stalker TJ, Cieslak M, Fan R, Boumsell L, Kumanogoh A, Kikutani H, et al Regulated surface expression and shedding support a dual role for semaphorin 4D in platelet responses to vascular injury. Proc Natl Acad Sci U S A 2007;104:1621–1626. 17244710 10.1073/pnas.0606344104 PMC1785259

[51] Charrin S, Jouannet S, Boucheix C, Rubinstein E. Tetraspanins at a glance. J Cell Sci 2014;127:3641–3648. 25128561 10.1242/jcs.154906

[52] Hemler ME. Tetraspanin proteins promote multiple cancer stages. Nat Rev Cancer 2014;14:49–60. 24505619 10.1038/nrc3640

[53] Min G, Wang H, Sun TT, Kong XP. Structural basis for tetraspanin functions as revealed by the cryo-EM structure of uroplakin complexes at 6-A resolution. J Cell Biol 2006;173:975–983. 16785325 10.1083/jcb.200602086 PMC2063921

[54] Zuidscherwoude M, Gottfert F, Dunlock VM, Figdor CG, van den Bogaart G, van Spriel AB The tetraspanin web revisited by super-resolution microscopy. Sci Rep 2015;5:12201. 26183063 10.1038/srep12201 PMC4505338

[55] Lammerding J, Kazarov AR, Huang H, Lee RT, Hemler ME. Tetraspanin CD151 regulates alpha6beta1 integrin adhesion strengthening. Proc Natl Acad Sci U S A 2003;100:7616–7621. 12805567 10.1073/pnas.1337546100 PMC164635

[56] Winterwood NE, Varzavand A, Meland MN, Ashman LK, Stipp CS. A critical role for tetraspanin CD151 in alpha3beta1 and alpha6beta4 integrin-dependent tumor cell functions on laminin-5. Mol Biol Cell 2006;17:2707–2721. 16571677 10.1091/mbc.E05-11-1042 PMC1474805

[57] Yang XH, Richardson AL, Torres-Arzayus MI, Zhou P, Sharma C, Kazarov AR, Andzelm MM, Strominger JL, Brown M, Hemler ME. CD151 accelerates breast cancer by regulating alpha 6 integrin function, signaling, and molecular organization. Cancer Res 2008;68:3204–3213. 18451146 10.1158/0008-5472.CAN-07-2949 PMC4764302

[58] Baldwin G, Novitskaya V, Sadej R, Pochec E, Litynska A, Hartmann C, Williams J, Ashman L, Eble JA, Berditchevski F. Tetraspanin CD151 regulates glycosylation of (alpha)3(beta)1 integrin. J Biol Chem 2008;283:35445–35454. 18852263 10.1074/jbc.M806394200

[59] Yang XH, Mirchev R, Deng X, Yacono P, Yang HL, Golan DE, Hemler ME. CD151 restricts the alpha6 integrin diffusion mode. J Cell Sci 2012;125:1478–1487. 22328509 10.1242/jcs.093963 PMC3336378

[60] Levy S. Function of the tetraspanin molecule CD81 in B and T cells. Immunol Res 2014;58:179–185. 24522698 10.1007/s12026-014-8490-7

[61] Junge HJ, Yang S, Burton JB, Paes K, Shu X, French DM, Costa M, Rice DS, Ye W. TSPAN12 regulates retinal vascular development by promoting Norrin- but not Wnt-induced FZD4/beta-catenin signaling. Cell 2009;139:299–311. 19837033 10.1016/j.cell.2009.07.048

[62] Barreiro O, Zamai M, Yanez-Mo M, Tejera E, Lopez-Romero P, Monk PN, Gratton E, Caiolfa VR, Sanchez-Madrid F. Endothelial adhesion receptors are recruited to adherent leukocytes by inclusion in preformed tetraspanin nanoplatforms. J Cell Biol 2008;183:527–542. 18955551 10.1083/jcb.200805076 PMC2575792

[63] Doyle EL, Ridger V, Ferraro F, Turmaine M, Saftig P, Cutler DF. CD63 is an essential cofactor to leukocyte recruitment by endothelial P-selectin. Blood 2011;118:4265–4273. 21803846 10.1182/blood-2010-11-321489

[64] Karamatic Crew V, Burton N, Kagan A, Green CA, Levene C, Flinter F, Brady RL, Daniels G, Anstee DJ. CD151, the first member of the tetraspanin (TM4) superfamily detected on erythrocytes, is essential for the correct assembly of human basement membranes in kidney and skin. Blood 2004;104:2217–2223. 15265795 10.1182/blood-2004-04-1512

[65] Nikopoulos K, Gilissen C, Hoischen A, van Nouhuys CE, Boonstra FN, Blokland EA, Arts P, Wieskamp N, Strom TM, Ayuso C, et al Next-generation sequencing of a 40 Mb linkage interval reveals TSPAN12 mutations in patients with familial exudative vitreoretinopathy. Am J Hum Genet 2010;86:240–247. 20159111 10.1016/j.ajhg.2009.12.016 PMC2820179

[66] Poulter JA, Ali M, Gilmour DF, Rice A, Kondo H, Hayashi K, Mackey DA, Kearns LS, Ruddle JB, Craig JE, et al Mutations in TSPAN12 cause autosomal-dominant familial exudative vitreoretinopathy. Am J Hum Genet 2010;86:248–253. 20159112 10.1016/j.ajhg.2010.01.012 PMC2820188

[67] van Zelm MC, Smet J, Adams B, Mascart F, Schandene L, Janssen F, Ferster A, Kuo CC, Levy S, van Dongen JJ, et al CD81 gene defect in humans disrupts CD19 complex formation and leads to antibody deficiency. J Clin Invest 2010;120:1265–1274. 20237408 10.1172/JCI39748 PMC2846042

[68] Goschnick MW, Lau LM, Wee JL, Liu YS, Hogarth PM, Robb LM, Hickey MJ, Wright MD, Jackson DE. Impaired “outside-in” integrin alphaIIbbeta3 signaling and thrombus stability in TSSC6-deficient mice. Blood 2006;108:1911–1918. 16720835 10.1182/blood-2006-02-004267

[69] Orlowski E, Chand R, Yip J, Wong C, Goschnick MW, Wright MD, Ashman LK, Jackson DE. A platelet tetraspanin superfamily member, CD151, is required for regulation of thrombus growth and stability in vivo. J Thromb Haemost 2009;7:2074–2084. 19740096 10.1111/j.1538-7836.2009.03612.x

[70] Haining EJ, Yang J, Tomlinson MG. Tetraspanin microdomains: fine-tuning platelet function. Biochem Soc Trans 2011;39:518–523. 21428931 10.1042/BST0390518

[71] Protty MB, Watkins NA, Colombo D, Thomas SG, Heath VL, Herbert JM, Bicknell R, Senis YA, Ashman LK, Berditchevski F, et al Identification of Tspan9 as a novel platelet tetraspanin and the collagen receptor GPVI as a component of tetraspanin microdomains. Biochem J 2009;417:391–400. 18795891 10.1042/BJ20081126 PMC2652832

[72] Tomlinson MG. Platelet tetraspanins: small but interesting. J Thromb Haemost 2009;7:2070–2073. 19740095 10.1111/j.1538-7836.2009.03613.x

[73] Uchtmann K, Park ER, Bergsma A, Segula J, Edick MJ, Miranti CK. Homozygous loss of mouse tetraspanin CD82 enhances integrin alphaIIbbeta3 expression and clot retraction in platelets. Exp Cell Res 2015;339:261–269. 26562164 10.1016/j.yexcr.2015.11.006

[74] Mangin PH, Kleitz L, Boucheix C, Gachet C, Lanza F. CD9 negatively regulates integrin alphaIIbbeta3 activation and could thus prevent excessive platelet recruitment at sites of vascular injury. J Thromb Haemost 2009;7:900–902. 19228283 10.1111/j.1538-7836.2009.03322.x

[75] Schroder J, Lullmann-Rauch R, Himmerkus N, Pleines I, Nieswandt B, Orinska Z, Koch-Nolte F, Schroder B, Bleich M, Saftig P. Deficiency of the tetraspanin CD63 associated with kidney pathology but normal lysosomal function. Mol Cell Biol 2009;29:1083–1094. 19075008 10.1128/MCB.01163-08 PMC2643809

[76] Arduise C, Abache T, Li L, Billard M, Chabanon A, Ludwig A, Mauduit P, Boucheix C, Rubinstein E, Le Naour F. Tetraspanins regulate ADAM10-mediated cleavage of TNF-alpha and epidermal growth factor. J Immunol 2008;181:7002–7013. 18981120 10.4049/jimmunol.181.10.7002

[77] Dornier E, Coumailleau F, Ottavi JF, Moretti J, Boucheix C, Mauduit P, Schweisguth F, Rubinstein E. TspanC8 tetraspanins regulate ADAM10/Kuzbanian trafficking and promote Notch activation in flies and mammals. J Cell Biol 2012;199:481–496. 23091066 10.1083/jcb.201201133 PMC3483123

[78] Haining EJ, Yang J, Bailey RL, Khan K, Collier R, Tsai S, Watson SP, Frampton J, Garcia P, Tomlinson MG. The TspanC8 subgroup of tetraspanins interacts with A disintegrin and metalloprotease 10 (ADAM10) and regulates its maturation and cell surface expression. J Biol Chem 2012;287:39753–39765. 23035126 10.1074/jbc.M112.416503 PMC3501075

[79] Xu D, Sharma C, Hemler ME. Tetraspanin12 regulates ADAM10-dependent cleavage of amyloid precursor protein. Faseb j 2009;23:3674–3681. 19587294 10.1096/fj.09-133462 PMC2775005

[80] Prox J, Willenbrock M, Weber S, Lehmann T, Schmidt-Arras D, Schwanbeck R, Saftig P, Schwake M. Tetraspanin15 regulates cellular trafficking and activity of the ectodomain sheddase ADAM10. Cell Mol Life Sci 2012;69:2919–2932. 22446748 10.1007/s00018-012-0960-2 PMC11114675

[81] Wong E, Maretzky T, Peleg Y, Blobel CP, Sagi I. The functional maturation of A disintegrin and metalloproteinase (ADAM) 9, 10, and 17 requires processing at a newly identified proprotein convertase (PC) cleavage site. J Biol Chem 2015;290:12135–12146. 25795784 10.1074/jbc.M114.624072 PMC4424348

[82] Jouannet S, Saint-Pol J, Fernandez L, Nguyen V, Charrin S, Boucheix C, Brou C, Milhiet PE, Rubinstein E. TspanC8 tetraspanins differentially regulate the cleavage of ADAM10 substrates, Notch activation and ADAM10 membrane compartmentalization. Cell Mol Life Sci 2016;73:1895–1915. 10.1007/s00018-015-2111-z PMC4819958 26686862

[83] Zhou J, Fujiwara T, Ye S, Li X, Zhao H. Downregulation of Notch modulators, tetraspanin 5 and 10, inhibits osteoclastogenesis in vitro. Calcif Tissue Int 2014;95:209–217. 24935633 10.1007/s00223-014-9883-2 PMC4139439

[84] Noy PJ, Yang J, Reyat JS, Matthews AL, Charlton AE, Furmston J, Rogers DA, Rainger GE, Tomlinson MG. TspanC8 tetraspanins and A disintegrin and metalloprotease 10 (ADAM10) interact via their extracellular regions: Evidence for distinct binding mechanisms for different TspanC8s. J Biol Chem 2016;291:3145–3157. 10.1074/jbc.M115.703058 PMC4751363 26668317

[85] Kuhn S, Koch M, Nubel T, Ladwein M, Antolovic D, Klingbeil P, Hildebrand D, Moldenhauer G, Langbein L, Franke WW, et al A complex of EpCAM, claudin-7, CD44 variant isoforms, and tetraspanins promotes colorectal cancer progression. Mol Cancer Res 2007;5:553–567. 17579117 10.1158/1541-7786.MCR-06-0384

[86] Zeiler M, Moser M, Mann M. Copy number analysis of the murine platelet proteome spanning the complete abundance range. Mol Cell Proteomics 2014;13:3435–3445. 25205226 10.1074/mcp.M114.038513 PMC4256495

[87] Burkhart JM, Vaudel M, Gambaryan S, Radau S, Walter U, Martens L, Geiger J, Sickmann A, Zahedi RP. The first comprehensive and quantitative analysis of human platelet protein composition allows the comparative analysis of structural and functional pathways. Blood 2012;120:e73–e82. 22869793 10.1182/blood-2012-04-416594

[88] Bergbold N, Lemberg MK. Emerging role of rhomboid family proteins in mammalian biology and disease. Biochim Biophys Acta 2013;1828:2840–2848. 23562403 10.1016/j.bbamem.2013.03.025

[89] Freeman M. The rhomboid-like superfamily: molecular mechanisms and biological roles. Annu Rev Cell Dev Biol 2014;30:235–254. 25062361 10.1146/annurev-cellbio-100913-012944

[90] Langosch D, Scharnagl C, Steiner H, Lemberg MK. Understanding intramembrane proteolysis: from protein dynamics to reaction kinetics. Trends Biochem Sci 2015;40:318–327. 25941170 10.1016/j.tibs.2015.04.001

[91] Adrain C, Zettl M, Christova Y, Taylor N, Freeman M. Tumor necrosis factor signaling requires iRhom2 to promote trafficking and activation of TACE. Science 2012;335:225–228. 22246777 10.1126/science.1214400 PMC3272371

[92] McIlwain DR, Lang PA, Maretzky T, Hamada K, Ohishi K, Maney SK, Berger T, Murthy A, Duncan G, Xu HC, et al iRhom2 regulation of TACE controls TNF-mediated protection against Listeria and responses to LPS. Science 2012;335:229–232. 22246778 10.1126/science.1214448 PMC4250273

[93] Siggs OM, Xiao N, Wang Y, Shi H, Tomisato W, Li X, Xia Y, Beutler B. iRhom2 is required for the secretion of mouse TNFalpha. Blood 2012;119:5769–5771. 22550345 10.1182/blood-2012-03-417949 PMC3382936

[94] Zettl M, Adrain C, Strisovsky K, Lastun V, Freeman M. Rhomboid family pseudoproteases use the ER quality control machinery to regulate intercellular signaling. Cell 2011;145:79–91. 21439629 10.1016/j.cell.2011.02.047 PMC3149277

[95] Issuree PD, Maretzky T, McIlwain DR, Monette S, Qing X, Lang PA, Swendeman SL, Park-Min KH, Binder N, Kalliolias GD, et al iRHOM2 is a critical pathogenic mediator of inflammatory arthritis. J Clin Invest 2013;123:928–932. 23348744 10.1172/JCI66168 PMC3561822

[96] Christova Y, Adrain C, Bambrough P, Ibrahim A, Freeman M. Mammalian iRhoms have distinct physiological functions including an essential role in TACE regulation. EMBO Rep 2013;14:884–890. 23969955 10.1038/embor.2013.128 PMC3807218

[97] Li X, Maretzky T, Weskamp G, Monette S, Qing X, Issuree PD, Crawford HC, McIlwain DR, Mak TW, Salmon JE, et al iRhoms 1 and 2 are essential upstream regulators of ADAM17-dependent EGFR signaling. Proc Natl Acad Sci USA 2015;112:6080–6085. 25918388 10.1073/pnas.1505649112 PMC4434755

[98] Blaydon DC, Etheridge SL, Risk JM, Hennies HC, Gay LJ, Carroll R, Plagnol V, McRonald FE, Stevens HP, Spurr NK, et al RHBDF2 mutations are associated with tylosis, a familial esophageal cancer syndrome. Am J Hum Genet 2012;90:340–346. 22265016 10.1016/j.ajhg.2011.12.008 PMC3276661

[99] Saarinen S, Vahteristo P, Lehtonen R, Aittomaki K, Launonen V, Kiviluoto T, Aaltonen LA. Analysis of a Finnish family confirms RHBDF2 mutations as the underlying factor in tylosis with esophageal cancer. Fam Cancer 2012;11:525–528. 22638770 10.1007/s10689-012-9532-8

[100] Brooke MA, Etheridge SL, Kaplan N, Simpson C, O’Toole EA, Ishida-Yamamoto A, Marches O, Getsios S, Kelsell DP. iRHOM2-dependent regulation of ADAM17 in cutaneous disease and epidermal barrier function. Hum Mol Genet 2014;23:4064–4076. 24643277 10.1093/hmg/ddu120 PMC4110483

[101] Blaydon DC, Biancheri P, Di WL, Plagnol V, Cabral RM, Brooke MA, van Heel DA, Ruschendorf F, Toynbee M, Walne A, et al Inflammatory skin and bowel disease linked to ADAM17 deletion. N Engl J Med 2011;365:1502–1508. 22010916 10.1056/NEJMoa1100721

[102] Maney SK, McIlwain DR, Polz R, Pandyra AA, Sundaram B, Wolff D, Ohishi K, Maretzky T, Brooke MA, Evers A, et al Deletions in the cytoplasmic domain of iRhom1 and iRhom2 promote shedding of the TNF receptor by the protease ADAM17. Sci Signal 2015;8:ra109. 26535007 10.1126/scisignal.aac5356 PMC7202466

[103] Maretzky T, McIlwain DR, Issuree PD, Li X, Malapeira J, Amin S, Lang PA, Mak TW, Blobel CP. iRhom2 controls the substrate selectivity of stimulated ADAM17-dependent ectodomain shedding. Proc Natl Acad Sci USA 2013;110:11433–11438. 23801765 10.1073/pnas.1302553110 PMC3710827

